# Heat shock protein 20 (HSP20) is a novel substrate for protein kinase D1 (PKD1)

**DOI:** 10.1002/cbf.3147

**Published:** 2015-10-06

**Authors:** Yuan Yan Sin, George S. Baillie

**Affiliations:** ^1^ Institute of Cardiovascular and Medical Sciences, CMVLSUniversity of GlasgowGlasgowUK

**Keywords:** HSP20, PKD1, PKA, peptide array, cardiac remodelling

## Abstract

Heat shock protein 20 (HSP20) has cardioprotective qualities, which are triggered by PKA phosphorylation. PKD1 is also a binding partner for HSP20, and this prompted us to investigate whether the chaperone was a substrate for PKD1. We delineate the PKD1 binding sites on HSP20 and show for the first time HSP20 is a substrate for PKD1. Phosphorylation of HSP20 by PKD1 is diminished by pharmacological or siRNA reduction of PKD1 activity and is enhanced following PKD1 activation. Our results suggest that both PKA and PKD1 can both phosphorylate HSP20 on serine 16 but that PKA is the most dominant. © 2016 The Authors. Cell Biochemistry and Function published by John Wiley & Sons, Ltd.

**List of Abbreviations**
HDAC: histone deacetylase
HSP20: heat shock protein 20
ISO: isoprenaline
PKA: cAMP‐dependent protein kinase A
PKD1: protein kinase D1
SDS–PAGE: sodium dodecyl sulfate–polyacrylamide gel electrophoresis

## Introduction

Heat shock protein 20 (HSP20), also referred to as P20 or HSPB6, is ubiquitously expressed in a number of tissues with the highest levels in the heart.^1^ HSP20 is inactive or partially active under physiological conditions and converts to an active phosphorylated state upon modulation by stress kinases.^2^, ^3^ The phosphorylation of HSP20 is thought to be a key step in certain neurohormonal signalling pathways and is particularly important in the myocardial β‐adrenergic signalling cascade where it acts as an intermediate in a variety of cardioprotective responses to injuries arising from β‐agonist induced hypertrophy and ischemia/reperfusion.^4^, ^5^ The cardioprotecive signalling pathways activated by phospho‐HSP20 stem from the promotion actin binding by the chaperone^6^ and the inhibition of P38, JNK,^7^ caspase 3 and NF‐KB pathways.^8^ The phosphorylation of HSP20 normally occurs in response to increased levels of cyclic nucleotide second messengers that activate kinases such as cAMP‐dependent protein kinase A (PKA) and cGMP‐dependent protein kinase G (PKG).^9^ To date, most assumptions about the significance of HSP20 phosphorylation have been made around serine 16, which is located within the sequence motif (RRXS), a characteristic consensus motif for both PKA and PKG.^10^, ^11^

Recently, we have identified protein kinase D1 (PKD1) as a novel partner for HSP20 using high‐density ProtoArray analysis.^12^ Investigation into the physiological function of the PKD–HSP20 interaction was facilitated by a novel disruptor peptide, which acted to disassemble the complex and reveal its role in pathological cardiac growth and cardiac remodelling. Being a major PKD isoform in the heart, PKD1 has been implicated in the phosphorylation of several sarcomeric proteins, such as cardiac troponin I (TnI), cardiac myosin binding protein C and telethonin to regulate myocardial contractility.^13^ PKD1 also regulates class II histone deacetylases (HDACs), which are known as pro‐hypertrophy transcription regulators.^14^ Notably, these putative PKD1 substrates commonly contain a well‐defined phosphorylation motif in the sequence (LXRXXS/T), where an aliphatic amino acid (Ile/Leu/Val) and a basic amino acid (Arg) are located at the −5 and −3 positions relative to the serine target site, respectively.^15^, ^16^ Here, we show for the first time that HSP20 is a novel substrate for PKD1 and that the putative phospho‐site is Ser16 (also the PKA and PKG sites).

## Materials and Methods

### Antibodies and reagents

Anti‐HSP20 antibody (#07‐490) was purchased from Millipore. Anti‐HSP20‐phospho‐Ser16 (#ab58522) and α‐tubulin (#ab‐18251) were purchased from Abcam. Anti‐PKD1 (#2052) and anti‐phospho‐PKD (auto‐phosphorylation site at Ser916, #2051) antibodies were purchased from Cell Signaling. Horseradish peroxidase‐conjugated goat anti‐rabbit and anti‐mouse secondary antibodies, and PKA inhibitor KT5720 were purchased from Sigma. Anti‐RACK1 antibody (#sc‐17754) was purchased from Santa Cruz. PKD1–HSP20 disruptor peptide (disr pept) (GRDVAIKIIDKLRFPTKQESQLRNE) and control peptide (cont pept) (GAAVAIKIIAKLRFPTKQESQLRNE) were synthesised by GenScript and included an N‐terminal stearoyl group (CH_3_(CH_2_)_16_COOH), making them cell permeable. PKD1 activator bryostatin 1 and inhibitor Go6976 were purchased from Calbiochem. For cell studies, peptides/compounds were dissolved in dimethyl sulfoxide (DMSO) (Sigma) and used at a final concentration of DMSO ≤ 0·1% (v v^−1^).

### SPOT synthesis of peptides and overlay experiments

This was performed as described by us in detail elsewhere.^17^

### Cell culture and transfection

Neonatal rat cardiomyocytes were cultured as previously described.^18^ DNA plasmid constructs used for transfection included V5‐HSP20 in pDEST vector and GFP‐PKD1 in pEF‐BOS vector. siRNA‐mediated PKD1 knockdown in cardiomyocytes was carried out using a siRNA transfection kit specific for rat PKD1 (Santa Cruz, #sc‐36260) according to the manufacturer's protocol.

### Western blotting

Cellular extract proteins were resolved by sodium dodecyl sulfate–polyacrylamide gel electrophoresis (SDS–PAGE) and transferred onto nitrocellulose membranes for Western blotting. Immunoreactive proteins were detected using appropriate antibodies and visualised by enhanced chemiluminescence detection (Pierce). Quantification of the band intensity was accomplished by densitometry using Quantity One 1‐D software (Bio‐Rad).

### In vitro phosphorylation of His‐HSP20

A range of purified His‐HSP20 concentration (0·5–2 µg) was incubated with 1·0 g ml^−1^ or without active PKD1 protein (Abcam) in PKD phosphorylation buffer [20 mM Tris–HCl; pH 7·5, 10 mM MgCl_2_, 0·5 mM CaCl_2_, 1 mM dithiothreitol (DTT), 0·2 mg ml^−1^ bovine serum albumin (BSA)] supplemented with 100 μM adenosine‐5′‐triphosphate (ATP) for 1 h at 30 °C with agitation. The phosphorylated protein was then subjected to SDS–PAGE and immunoblotted with an anti‐phospho‐HSP20 (Ser 16) antibody.

### In vitro phosphorylation of peptide array

Peptide arrays corresponding to the entire sequence of HSP20 (25mers, sequentially shifted by five amino acids) were constructed as published previously^5^. Phosphorylation of HSP20 peptide array was carried out to identify phosphorylation site(s) by PKD1. Briefly, the peptide array membrane was soaked in 100% ethanol to activate it and washed with Tris‐buffered saline and Tween 20, followed by overnight blocking in 5% phosphoblocker containing 0·5 mM DTT and 1 mM ATP at 4 °C. The membrane was then reacted with PKD1 phosphorylation buffer (20 mM Tris–HCl; pH 7·5, 10 mM MgCl_2_, 0·5 mM CaCl_2_, 1 mM DTT, 0·2 mg ml^−1^ BSA, 1 mM ATP) supplemented with 10 µg of active PKD1 (Abcam) and 5 μCi (0·185 MBq) of [γ‐^32^P] ATP EasyTides (Perkin Elmer, UK) for 30 min at 37 °C with gentle agitation. After the phosphorylation reaction, the membrane was washed extensively with 1 M NaCl followed by 5% H_3_PO_4_ and finally ethanol. The membrane was air dried and placed in intensifying screen cassette (Kodak BioMax MS, Carestream Health, Inc.) for a week before developing using the Kodak® X‐Omat Model 2000 processor.

### Statistical analysis

Values are presented as mean ± SEM from at least three independent experiments. Statistical significances between groups were determined by the use of Student's *t*‐test or one‐way ANOVA tests followed by post hoc Tukey's tests. Values were considered significant if *p* < 0·05. Where representative immunoblots were shown, similar data were obtained *n* ≥ 3 times.

## Results

### HSP20 phosphorylation on serine 16 is influenced by PKD1 activity

**Figure 1:**
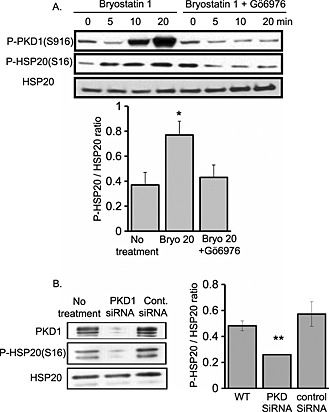
The effect of PKD1 activity on HSP20 phosphorylation. (A) HEK293 cells were treated with bryostatin 1 (10 nM) alone or with Go6976 (20 nM) for the indicated times. PKD1 activation and HSP20 phosphorylation were monitored via immunoblotting with anti‐phospho‐PKD1 (Ser916), which identified a band of approximately 100 kDa and anti‐phospho‐HSP20 (Ser16) antibodies, which identified a band of approximately 20 kDa. Anti‐HSP20 antibody was used as loading control, which identified a band of approximately 20 kDa. (B) siRNA‐mediated knockdown of PKD1 (but not control siRNA) attenuates HSP20 phosphorylation. The phosphorylation state (serine 16) was monitored following PKD1 silencing. *p < 0·05, **p < 0·01

As a sequence analysis of HSP20 showed it to contain a suitable consensus for PKD1 phosphorylation (S^10^W**L**R**R**X**S**APLP^20^), we sought to evaluate whether the heat shock protein could be phosphorylated by PKD1. We used two experimental approaches to examine the effect of PKD1 activity on the phosphorylation level of HSP20 at serine 16. Firstly, we utilised pharmacological inhibition/activation of the kinase. Cells were treated with activator bryostatin 1 (10 nM)^19^ or inhibitor Go6976 (20 nM).^20^ PKD1 activation was then assessed using an anti‐phospho‐PKD1 (auto‐phosphorylation site at Ser916) antibody that detects only the active form of the kinase.^21^ Our results showed that PKD1 activation was significantly elevated following stimulation with bryostatin 1 (*p* < 0·05) and diminished significantly (*p* < 0·01) after treatment with Go6976. Interestingly, the phosphorylation of HSP20 increased along with activation of PKD1 and converse inhibition of PKD1 decreased HSP20 phosphorylation (Figure 1A). Secondly, we undertook a loss‐of‐function approach to confirm the direct effect of PKD1 activation on HSP20 phosphorylation in an endogenous cell system. The transfection of neonatal rat cardiomyocytes with PKD1 siRNA markedly suppressed endogenous PKD1 expression, whereas non‐specific control siRNA had no effect (Figure 1B). In a correlation with PKD1 silencing, there was a profound reduction in the phosphorylation level of HSP20 (Ser16). This reduction in phospho‐HSP20 was not a result of fluctuating total endogenous HSP20 expression, which remained unchanged following PKD1 silencing (Figure 1B). Taken together, these data suggest that HSP20 phosphorylation at serine 16 can be influenced by PKD1 activity.

### Mapping PKD binding and phosphorylation sites on HSP20

**Figure 2:**
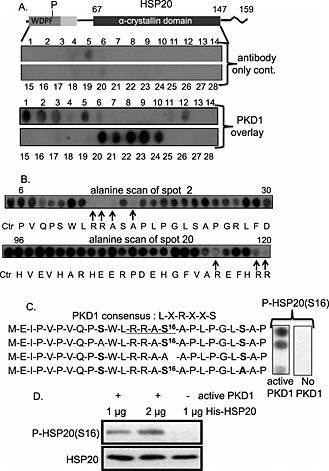
Identification of PKD1–HSP20 interaction and phosphorylation sites. (A) HSP20 is shown schematically with phosphorylation site (P), WDPF domain (dark shaded area), conserved region (light shaded area) and α‐crystallin domain (black area). Peptide array analysis identifies PKD1 binding sites on HSP20. (B) The key residues involved in the HSP20–PKD1 interaction are delineated using alanine scanning peptide arrays where each residue is sequentially substituted by alanine (or aspartate if residue is alanine). (C) *In vitro* phosphorylation assay on peptide array using [γ‐^32^P‐ATP] revealed that HSP20 is phosphorylated at Ser16 by PKD1. (D) *In vitro* kinase assay (cold) using purified His‐HSP20 and PKD1 active protein further verified HSP20 phosphorylation by PKD1

In an attempt to map the binding site of PKD1 on full‐length HSP20, we used peptide array, a technique previously utilised by us to pinpoint the interaction sites between HSP20 and PDE4.^5^ Results revealed overlapping binding regions in the WDPF and α‐crystallin domains (Figure 2A). Alanine scanning peptide arrays of residues 6–30 and 96–120 identified a number of key amino acids that are potentially involved in the HSP20–PKD1 interaction (Figure 2B). Specifically, in an alanine substituted peptide array encompassing amino acids P^6^‐D^30^ of HSP20, a loss of binding upon substitution of Arg^11,12^ with alanine and Ala^13,15^ with aspartic acid was observed. Interestingly, these amino acids form the domain surrounding Ser16 (RRApSAP), a site which is similar to the phospho‐motif of the AGC kinase subfamily (PKA, PKG and PKC).^7^ An *in vitro* phosphorylation assay carried out on HSP20 peptide arrays using [γ‐^32^P‐ATP] revealed an optimal putative PKD1 phosphorylation site at Ser16 (Figure 2C). Interestingly, this site falls within the minimal PKD recognition motif of LXRXXS, where an arginine is normally located at the −3 position. As reported previously, well‐known substrates of PKD1 such as HSP27, HDAC5, and cTnI commonly conform perfectly to this phosphorylation motif as identified through combinatorial peptide libraries.^13^, ^14^, ^16^, ^22^ Additionally, a cold *in vitro* kinase assay was carried out using recombinant purified His‐HSP20 protein and PKD1 active protein and then probed with HSP20 phospho‐Ser16 antibody to validate the specificity of the phosphorylation site. Phospho‐bands were detected when active PKD1 was added. No appreciable immunoreactivity was evident when the assay mix was devoid of PKD1 (Figure 2C). These data support the notion that PKD1 binds directly to HSP20 in order to phosphorylate it at serine 16. Although no other conventional PKD sites (apart from serine 16) exist within the HSP20 sequence, we cannot rule out the possibility that PKD1 has the ability to phosphorylate HSP20 at other sites.

### Disruption of the PKD1–HSP20 complex reduces HSP20 phosphorylation

**Figure 3:**
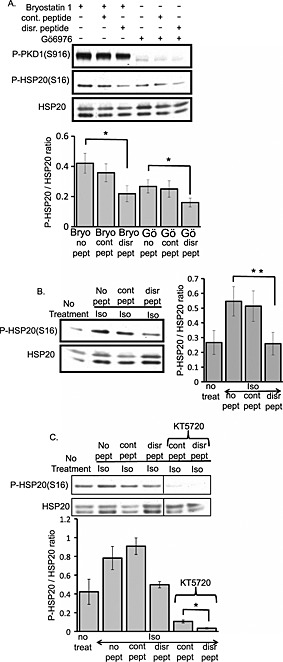
The effect of PKD1–HSP20 interaction on HSP20 phosphorylation. (A) Cardiomyocytes were subjected to compound treatment as indicated [bryostatin 1 (10 nM), Go6976 (20 nM), control peptide (10 μM) and PKD1–HSP20 disruptor peptide (10 μM)]. Protein lysates were subjected to immunoblotting with antibodies that recognise phospho‐PKD (Ser916) and phospho‐HSP20 (Ser16). Anti‐HSP20 antibody was used as loading control. (B) The disruption of PKD1–HSP20 interaction decreased HSP20 phosphorylation in ISO‐stimulated cardiomyocytes. Cells were pre‐treated with control or disruptor peptide (10 μM) prior to 24 h ISO (10 μM) stimulation. Lysates were immunoblotted with anti‐phospho‐HSP20 (Ser16) with anti‐HSP20 as loading control. (C) The inhibition of PKA activity (KT5720 1 μM) and disruption of PKD1–HSP20 interaction greatly decreased HSP20 phosphorylation. Immunoblots showing HSP20 phosphorylation level with respect to different treatments (upper panel). Quantification of immunoband intensities was determined by densitometry and was presented as fold ratio of phosphorylation level normalised to HSP20 ± SEM of three experiments on different cardiomyocytes preparations. *p < 0·05, **p < 0·01

As our data suggest a direct interaction between HSP20 and PKD1, we were keen to determine whether the disruption of PKD1–HSP20 interaction affects levels of HSP20 phosphorylation. Using a cell permeable peptide disruptor of the PKD1–HSP20 complex previously characterised in both *in vitro* and *in vivo* studies,^12^ we observed a reduction in HSP20 phosphorylation following peptide treatment (but not control peptide treatment) (Figure 3A). This reduction was not due to a variation in cardiomyocytes total PKD1 activity as measured by phospho‐PKD1 (Ser916) level, suggesting that PKD1–HSP20 interaction is required for HSP20 phosphorylation. In light of the involvement of HSP20 phosphorylation in mediating cardiac responses in cultured cardiomyocytes, we also examined the effect of PKD–HSP20 complex disruption on HSP20 phosphorylation in isoprenaline (ISO)‐stimulated cardiomyocytes. Once again, the disruptor peptide but not the control peptide caused a significant decrease in phospho‐HSP20 levels supporting the notion that a decrease in HSP20 phosphorylation level can be caused by the disruption of PKD1–HSP20 interaction without affecting PKD1 activity (Figure 3B). As HSP20 can be phosphorylated on serine 16 by both PKA and PKD1, we investigated whether their combined input is needed for maximal phosphorylation. Gratifyingly, treatment with a PKA‐selective inhibitor, KT5720, resulted in nearly 80% reduction in HSP20 phosphorylation, and this was further diminished upon disruption of PKD1–HSP20 interaction in cardiomyocytes (Figure 3C). These data are in agreement with those of the previous work, which suggests that PKA is the dominant mediator of HSP20 phosphorylation,^23^ although it is clear that PKD1 also has a role to play in this regard. We also note that the basal phosphorylation of HSP20 is almost ablated following PKA inhibition, suggesting that under resting conditions, HSP20 is phosphorylated by a pool of PKA that can be activated by the action of basally active adenylate cyclase.

## Discussion

In this study, we report for the first time that HSP20 is a substrate for PKD1. In identifying the binding sites and phosphorylation site for PKD1 on HSP20, we suggest that PKD1 binds directly to the heat shock protein to enable phosphorylation at a PKD consensus site, which contains serine 16. Indeed, the complex between these proteins has previously been reported.^12^ However, in addition to its role in trafficking PKD1, we now show that HSP20 acts as a substrate for the kinase. Interestingly, we identify the PKD1 phospho‐site as the one also modified by PKA and PKG.^10^, ^11^ Previous work has shown that PKD1 can phosphorylate the same sites as PKA on TnI^13^, ^24^; therefore, it is unsurprising that PKD1 is also capable of phosphorylating other PKA targets such as HSP20 as seen in this study.

The results outlined earlier are of particular interest in light of previous research, which has described increased PKA phosphorylation of HSP20 as a cardioprotective mechanism following sustained β‐adrenergic stimulation.^23^ This is in contrast to our recent findings, which suggest that cardioprotection is induced by disruption of PKD1–HSP20 complex,^12^ an act that should result in a reduction of HSP20 phosphorylation (as reported in this study). There are examples in the literature where different kinases can elicit opposite effects despite phosphorylating the same residue.^25^, ^26^ One possible explanation is that the functional roles of HSP20 are associated with its phospho‐Ser16 status resulting from differential kinase activation within ‘sub‐pools’ of HSP20. This notion is supported by our finding that the combined effect of PKA inhibition and disruption of PKD1–HSP20 complex on HSP20 phosphorylation was additive. PKA could function in concert with PKD1 to elicit signalling changes in response to hypertrophic stimuli. Although the functional role of PKD1 phosphorylation of HSP20 is not a subject of this paper, we interpret these results to suggest that HSP20 phosphorylation at Ser16 initiates cardioprotection in a kinase‐dependent manner, with PKA being the dominant kinase with respect to HSP20 phosphorylation. In this regard, HSP20 may serve as a ‘regulatory hotspot’ for two opposing signalling cues that modulate cardiac response. The balance between PKA and PKD1 activation is likely to prescribe the consequence of a hypertrophic response. Nevertheless, how HSP20 discriminates between PKA and PKD1 in different settings is still unclear. Further work to elucidate the mechanistic details underpinning the crosstalk between different kinase‐mediated cellular responses would shed new light on the role(s) of HSP20 in cardiac hypertrophy.

## Author Contributions

Y. Y. S. and G. S. B. conceived the study, designed the experiments, and wrote the manuscript. Y. Y. S. performed the experiments.
